# Corncob structures in dental plaque reveal microhabitat taxon specificity

**DOI:** 10.1186/s40168-022-01323-x

**Published:** 2022-09-05

**Authors:** Viviana Morillo-Lopez, Alexandra Sjaarda, Imon Islam, Gary G. Borisy, Jessica L. Mark Welch

**Affiliations:** 1 Josephine Bay Paul Center for Comparative Molecular Biology and Evolution, Marine Biological Laboratory, Woods Hole, MA 02543 USA; 2 Present Address: Department of Microbiology, The Forsyth Institute, Cambridge, MA 02139 USA

**Keywords:** Oral microbiome, Biogeography, Fluorescence *in situ* hybridization, FISH, Microscopy, Imaging, Corncob, Hedgehog, Microbial ecology

## Abstract

**Background:**

The human mouth is a natural laboratory for studying how bacterial communities differ across habitats. Different bacteria colonize different surfaces in the mouth—teeth, tongue dorsum, and keratinized and non-keratinized epithelia—despite the short physical distance between these habitats and their connection through saliva. We sought to determine whether more tightly defined microhabitats might have more tightly defined sets of resident bacteria. A microhabitat may be characterized, for example, as the space adjacent to a particular species of bacterium. Corncob structures of dental plaque, consisting of coccoid bacteria bound to filaments of *Corynebacterium* cells, present an opportunity to analyze the community structure of one such well-defined microhabitat within a complex natural biofilm. Here, we investigate by fluorescence in situ hybridization and spectral imaging the composition of the cocci decorating the filaments.

**Results:**

The range of taxa observed in corncobs was limited to a small subset of the taxa present in dental plaque. Among four major groups of dental plaque streptococci, two were the major constituents of corncobs, including one that was the most abundant *Streptococcus* species in corncobs despite being relatively rare in dental plaque overall. Images showed both *Streptococcus* types in corncobs in all individual donors, suggesting that the taxa have different ecological roles or that mechanisms exist for stabilizing the persistence of functionally redundant taxa in the population. Direct taxon-taxon interactions were observed not only between the *Streptococcus* cells and the central corncob filament but also between *Streptococcus* cells and the limited subset of other plaque bacteria detected in the corncobs, indicating species ensembles involving these taxa as well.

**Conclusions:**

The spatial organization we observed in corncobs suggests that each of the microbial participants can interact with multiple, albeit limited, potential partners, a feature that may encourage the long-term stability of the community. Additionally, our results suggest the general principle that a precisely defined microhabitat will be inhabited by a small and well-defined set of microbial taxa. Thus, our results are important for understanding the structure and organizing principles of natural biofilms and lay the groundwork for future work to modulate and control biofilms for human health.

Video Abstract

**Supplementary Information:**

The online version contains supplementary material available at 10.1186/s40168-022-01323-x.

## Background

Microbial community complexity in the human mouth depends on the scale on which it is assessed. The mouth as a whole has some 700 resident microbial taxa [1–3]. This large set of microbes is subdivided into smaller sets specialized for the different habitats within the mouth, such as dental plaque, tongue dorsum, and buccal mucosa [4–9]. Some of the microbes within dental plaque are specialized for subgingival rather than supragingival habitats [8, 10]; others are rare in healthy plaque but abundant in disease states such as caries or periodontal disease [11]. Investigating spatial organization at sub-millimeter scales using imaging, we have discovered organized consortia tens to hundreds of micrometers in diameter both in supragingival dental plaque [12] and on the tongue dorsum [13]. Each of these consortia contained a subset of the taxa that were found at the site overall. These findings raise the question: is it possible that the apparent enormous complexity of microbial communities is a consequence of combining many distinct habitats in a single sample, and that the more precisely a habitat can be defined, the smaller the number of microbes that grow there?

The habitat for a microbe is defined in large part by the other microbes located within a radius of a few micrometers to tens of micrometers. Short-range interactions between taxa shape the physiology of individual microbes and of microbial communities as a whole. Microbes exude metabolites that stimulate or inhibit growth of neighboring microbes [14–16] or cause them to alter their metabolism [16–18]. These interactions are strongest at distances of only a few micrometers, particularly in situations where fluid flow can rapidly attenuate the concentration of a metabolite [19] or within dense aggregations of microbes in which the distance over which a metabolite is available depends on the rates at which it is secreted and taken up by neighboring microbes [20]. Microbial surfaces also present binding sites to which other microbes may adhere and which thereby enable direct taxon-taxon interaction [21] and permit the localization of a microbe into a favorable habitat. For these reasons, the local neighborhood and nearest-neighbor relationships of a microbe play a major role in defining its habitat.

Corncob structures of dental plaque present an opportunity to analyze a well-defined microhabitat within the full complexity of a natural microbial community. In an otherwise amorphous mass of plaque bacteria, corncobs are discrete, readily recognizable structures characterized by direct physical interaction between filaments and cocci, as shown first by light microscopy [22, 23] and subsequently by electron microscopy [24–26]. Microdissection of corncobs followed by cultivation [27] identified the filament as *C. matruchotii* and the cocci as *Streptococcus sanguis* (subsequently renamed *S. crista* [28], then *S. cristatus* [29]). The potential involvement of additional partners was suggested by reconstruction experiments showing that *S. sanguis* cocci could associate with *Fusobacterium nucleatum* to form corncob-like structures in vitro [30]. However, the relationship of these corncob-like, cocci-filament associations to the structures previously identified as corncobs in dental plaque was not established. Our imaging confirmed the presence in natural plaque of a filamentous *Corynebacterium* core decorated by cocci of genus *Streptococcus* but also revealed additional participants in these corncobs, including members of the genus *Porphyromonas* and the family *Pasteurellaceae* [12]. These observations suggest that the species composition of corncobs is simple enough to be tractable but complex enough to offer insight into the rules governing community assembly within a natural microbiome.

To investigate the degree of selectivity of the corncob microhabitat, and the site-specificity of its component taxa, we focused on the healthy human mouth and on species of the genus *Streptococcus*. Among the genera of oral bacteria, *Streptococcus* stands out for its high abundance throughout the mouth, with multiple species that are abundant and prevalent in healthy dental plaque. Here, we investigate whether corncob structures in dental plaque represent a species ensemble involving a single species of *Streptococcus*, or whether more than one *Streptococcus* species can associate with *Corynebacterium* and with the other cocci in corncobs. Our results indicate that the corncob microhabitat can be occupied apparently interchangeably by more than one species but not all species of plaque *Streptococcus*. To our knowledge, this is the first report identifying at the species level direct spatial interaction involving more than two partners in a natural biofilm. Our results are thus important for learning how such biofilms are constructed and eventually how to manipulate their composition, particularly in the human microbiome. Notwithstanding their apparently interchangeable positions, two types of corncob-forming *Streptococcus* coexisted in all donors sampled, suggesting either that they occupy different ecological niches or that mechanisms exist that maintain redundancy and diversity in this host-microbiome system.

## Methods

### Sample collection and preparation

Samples of supragingival dental plaque from 14 healthy donors older than 18 years old were collected using toothpicks. All donors provided written informed consent. Donors were asked to provide information on their diet (vegetarian or meat-eating) and current tobacco use; all 14 donors reported a meat-eating or non-vegetarian diet and no current tobacco use. Donors were asked not to perform oral hygiene for at least 12 to 24 h prior to sample collection. Samples were fixed in 2% paraformaldehyde (Electron Microscopy Sciences) in 1× phosphate buffered saline (PBS), with 4 h of incubation on ice. Samples were then washed 3 times with 10 mM Tris-HCl pH 7.5 allowing settling by gravity rather than centrifugation between each wash to minimize disruption of structures in the dental plaque. Samples were stored in a 1:1 mixture of 96% ethanol and 10 mM Tris-HCl pH 7.5 at −20 °C until use.

### Probe design and testing

We designed FISH probes targeting species of *Streptococcus* that are abundant in supragingival plaque and potentially involved in the formation of corncobs; one probe targeted *S. gordonii*, one targeted *S. cristatus*, one targeted *S. mitis* and its close relatives *S. infantis* and *S. oralis*, and one targeted both *S. cristatus* and the *S. mitis/oralis/infantis* group (Additional file 1). Probe sites were selected by inspecting an alignment of 16S rRNA sequences of oral *Streptococcus* species extracted from sequences deposited in the expanded Human Oral Microbiome Database (eHOMD) [31]. Candidate oligonucleotide probes were tested for specificity in silico using mathFISH [32] to calculate predicted free energy of hybridization and predicted hybridization efficiency on probe sites of target taxa and non-target taxa. If the calculated hybridization efficiency on the target taxon in 20–30% formamide was low, the probes were lengthened by several nucleotides.

**Fig. 1:**
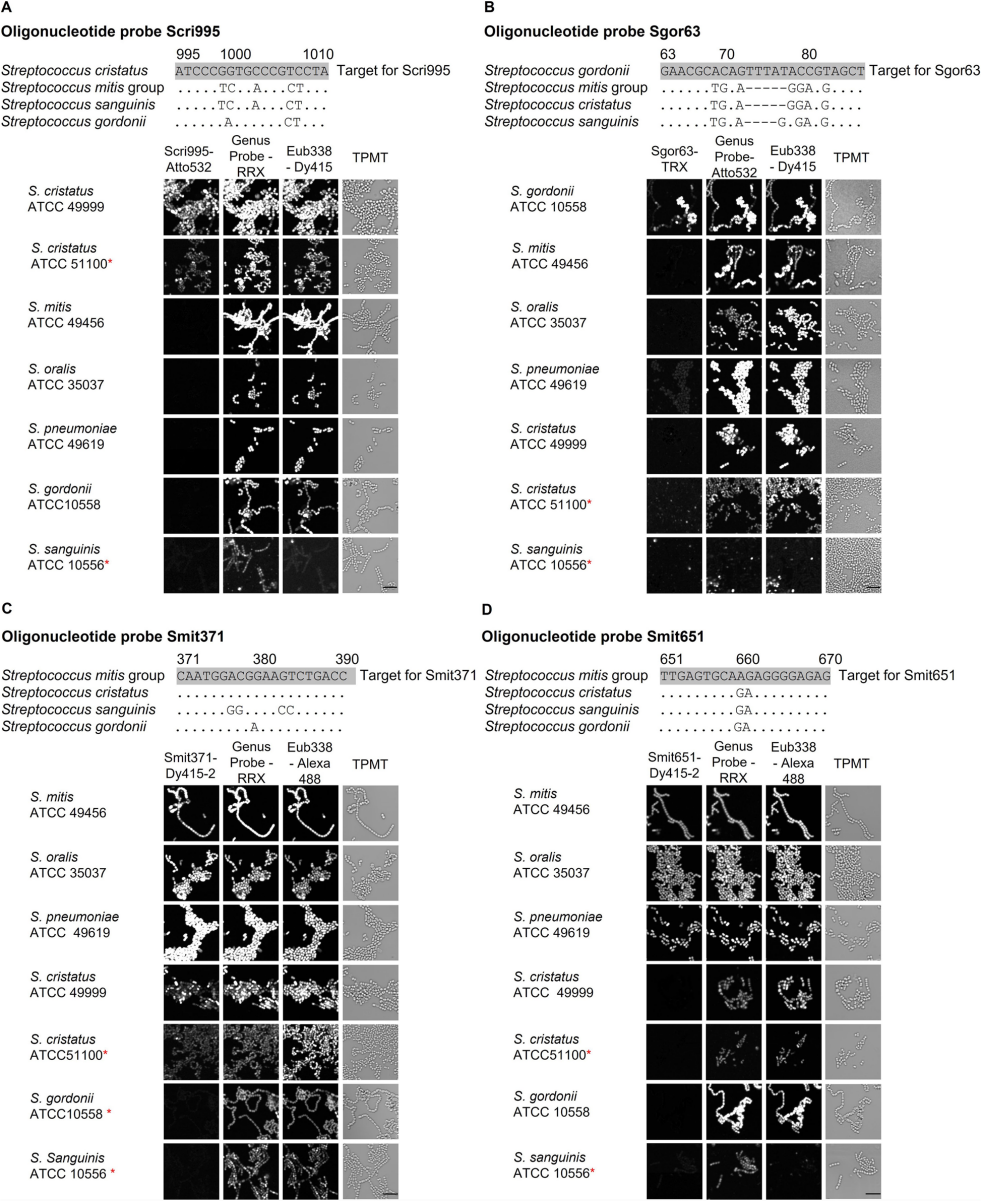
Validation of new oligonucleotide probes targeting subsets of the genus *Streptococcus*. Each newly designed probe was hybridized to pure cultures simultaneously with existing probes targeting genus *Streptococcus* and most Bacteria. 15 pure cultures were hybridized; 7 are shown here and the remaining 8 are shown in Additional file 1. **A** Probe Scri995 targeting *S. cristatus*. **B** Probe Sgor63 targeting *S. gordonii*. **C** Probe Smit371 targeting *S. mitis* and its close relatives and *S. cristatus*. **D** probe Smit651 targeting *S. mitis* and its close relatives. For each probe set, image acquisition and linear unmixing were carried out under the same conditions using Zeiss ZEN software. Images were imported into FIJI and the range of display intensities was kept constant for each fluorophore (each column in the figure); for cultures where the fluorescence was dim in all channels, the display range was then additionally adjusted by a constant factor for all images in the row in order to improve visibility of cells; these rows are marked (*). All probes hybridized with their expected targets and showed negligible cross-hybridization to unexpected targets. A full list of oral *Streptococcus* species and their matches and mismatches to each probe is shown in Additional file 1. (RRX: Rhodamine Red X; TRX: Texas Red X). Scale bars = 5 micrometers

The abbreviated probe names indicate the major targeted taxon and the position of the probe target site along the 16S rRNA; probe Scri995 targets *S. cristatus*, Sgor63 targets *S. gordonii*, Smit651 targets the *S. mitis*/*oralis*/*infantis* group, and Smit371 targets the *S. mitis*/*oralis*/*infantis* group and *S. cristatus*. Target sequences are shown in Fig. 1 together with an alignment of the corresponding sequences from four major supragingival plaque *Streptococcus* spp. The alignments also indicate some potential off-target interactions: Scri995 and Smit371 are expected to hybridize with *S. sinensis,* and Sgor63 with *S. anginosus*. However, oligotyping has shown that both species are rare in healthy supragingival plaque [7, 9] and thus unlikely to be a source of ambiguity. The *S. mitis* group, which we define as species nearly identical to *S. mitis* in 16S rRNA gene sequence, includes *S. infantis*, *S. oralis*, and *S. pneumoniae.* A complete description of the expected specificity of these probes on all *Streptococcus* spp. in eHOMD is given in Additional file 1.

Fluorophore-labeled oligonucleotide probes were custom-synthesized (Biomers.net, Ulm/Donau, Germany) and tested experimentally for specificity and sensitivity on 15 pure cultures of streptococci as well as additional taxa. FISH was carried out on *S. cristatus* (3 strains), *S. mitis* (2 strains), *S. gordonii*, *S. vestibularis*, *S. sanguinis, S. parasanguinis*, *Porphyromonas gingivalis*, *Aggregatibacter aphrophilus*, *Corynebacterium durum*, and *C. matruchotii* to validate the specificity and sensitivity of each set of probes. A complete list of strains used to test the specificity and sensitivity of the probes is shown in Additional file 2.

### Probe sets

To image corncobs, we used 3 sets of probes (Additional file 3) targeting bacteria representing some of the most abundant taxa in the supragingival plaque: family *Pasteurellaceae* (genera *Haemophilus* and *Aggregatibacter*)*,* genera *Streptococcus, Corynebacterium*, and *Porphyromonas*, and species *S. cristatus*, *S. gordonii*, and *S. mitis/oralis/infantis* and close relatives [7, 12]. Probe sequences and the composition of probe sets are shown in Additional file 3. The Pasteurellaceae, *Corynebacterium*, and *Porphyromonas* probes are described in Mark Welch et al. 2016 [12]; the *Corynebacterium* genus probe targets both major oral *Corynebacterium* species, *C. matruchotii* and *C. durum*, and the *Porphyromonas* probe targets the *P. gingivalis* group including *P. catoniae* and *P. pasteri* but not *P. endodontalis*.

### FISH

Approximately 100 μl of fixed dental plaque in 50% ethanol/10 mM Tris pH 7.5 was spread onto Gold Seal UltraStick adhesion slides (ThermoFisher) and allowed to air-dry immediately before FISH. One hundred microliters of hybridization buffer (0.9 M NaCl, 20 mM Tris-HCl pH 7.5, 0.01% SDS, 20% Hi-Di formamide (ThermoFisher)) containing 2 pmol/μl of each probe were added. Each slide was covered with a 22 × 40 mm cover slip and incubated in a humid chamber at 46 ^o^C for 4 h. Slides were washed once with wash buffer (215 mM NaCl, 20 mM Tris-HCl pH 7.5, 5 mM EDTA), incubated 15 min at 48 ^o^C, and rinsed with cold water. Slides were mounted in ProLong Gold antifade and covered with a 22 × 50 mm #1.5 coverslip. The same protocol was used for hybridization on pure cultures except that 10 μl of a fixed culture was used.

### Image acquisition

Spectral images were acquired using either a Zeiss 780 or Zeiss 880 laser scanning confocal microscope equipped with a 32-anode spectral detector and a 40×, 1.4 NA Plan-Apochromat objective. Samples were imaged using 633, 561, 488, and 405 nm excitation wavelengths. Images of pure cultures were acquired using the same imaging conditions as the plaque samples. Images were acquired at a resolution of 9.64 pixels/μm (2048 × 2048 pixels and 212.55 × 212.55 μm).

### Image analysis

Reference spectra for each fluorophore used in this study were measured on *Leptotrichia buccalis* cells labeled with the Eub338 probe conjugated to the appropriate fluorophore. The acquired images were processed by applying a median filter with a 3 × 3 kernel, followed by linear unmixing in the Zeiss ZEN Black software using the respective reference spectra. Unmixed images were imported into FIJI [33] to generate maximum intensity projections of *z*-stack images and to select and false-color unmixed channels for overlay images using the Image5D plug-in.

## Results

### Dental plaque hedgehogs contain both single-taxon and mixed corncobs

The genus *Streptococcus* is species-rich, with 36 oral or potentially oral species recognized in the expanded Human Oral Microbiome Database (eHOMD) [31]. Of these, four subgroups are abundant in dental plaque: the *S. mitis/oralis/infantis* group, *S. sanguinis*, *S. gordonii*, and *S. cristatus* [7]. To investigate spatial organization of *Streptococcus* species in corncobs, we designed FISH probes targeting subgroups of *Streptococcus* species and applied them to supragingival plaque sampled from healthy volunteers. We designed probes to differentiate among groups of species so that collectively the probes could generate a distinctive hybridization pattern for *S. cristatus*, *S. gordonii*, and the *S. mitis* group including *S. mitis*, *S. oralis*, and *S. infantis*. We tested each probe for effectiveness and specificity by hybridizing it with pure cultures representative of target and non-target taxa. For comparison, each culture was also hybridized simultaneously with a universal bacterial probe and a probe for the genus *Streptococcus*. The target taxa showed the expected probe signals (Fig. 1, Additional file 1).

Having established the specificity of these new species and subgroup-level probes, we combined them with existing probes targeting genus- and family-level taxa to create probe sets to illuminate corncob structure. In addition to *Streptococcus* at the genus and species level, the probe sets targeted the other taxa previously demonstrated to participate in corncobs: the genera *Corynebacterium* and *Porphyromonas* and the family *Pasteurellaceae* [12]. We employed probes in different combinations, using different fluorophores, to ensure robustness of results to the details of the probe set composition. A detailed description of each probe set and its validation on pure cultures is presented in Additional files 3 and 4.

**Fig. 2:**
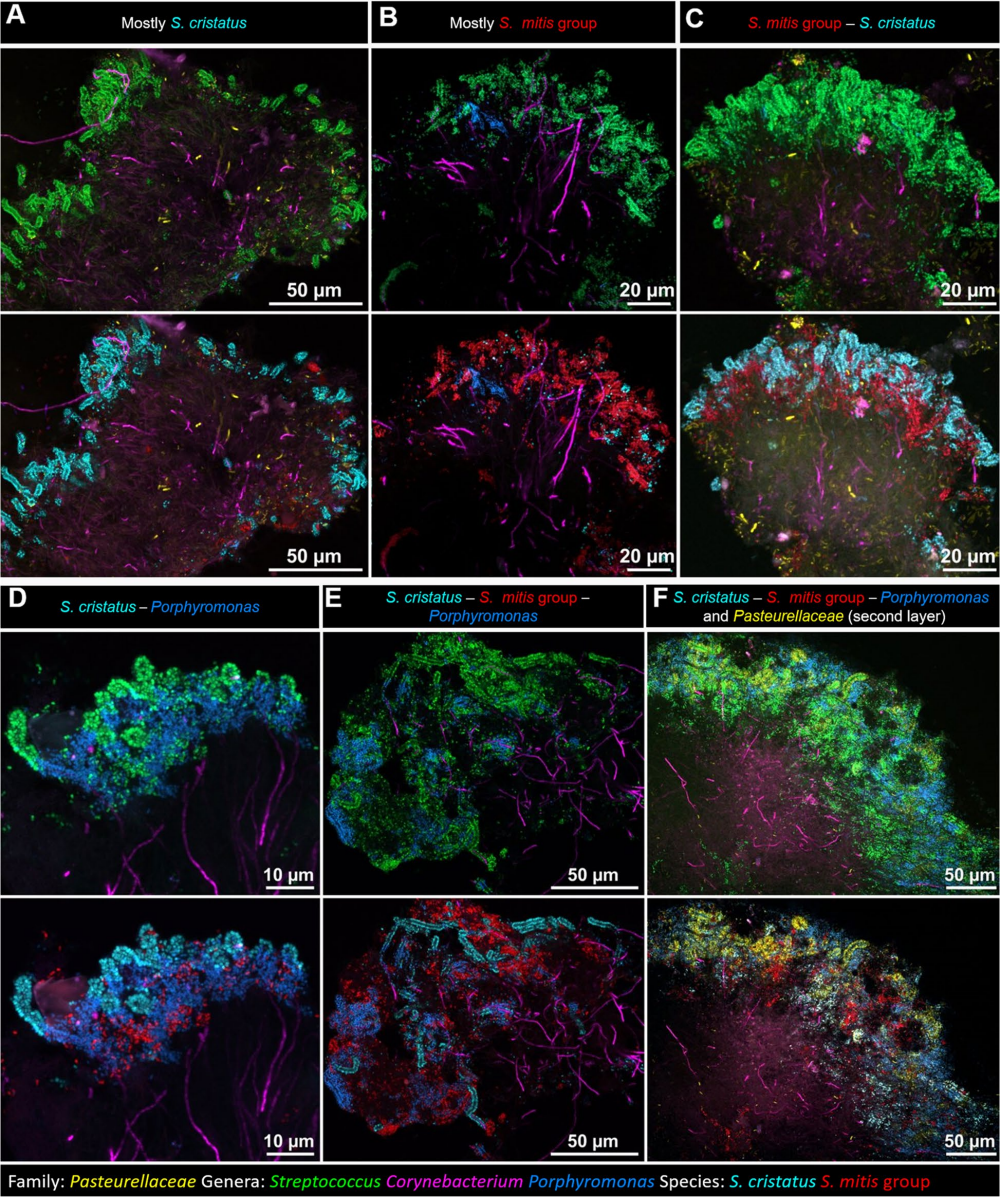
Dental plaque hedgehogs contain corncobs of mixed composition. Hedgehog structures are identified in plaque by the presence of a cluster of *Corynebacterium* filaments with corncobs at the periphery. Family- and genus-level probes (top in each pair of images) show corncobs composed of cells of genera *Streptococcus* (green), *Porphyromonas* (blue), and *Corynebacterium* (magenta) and family *Pasteurellaceae* (yellow). Species-level probes (bottom in each pair of images) show that the *Streptococcus* population in a given hedgehog can contain mostly *S. cristatus* (cyan) (**A**), mostly *S. mitis/oralis/infantis* (red) (**B**), or a mixture of both *S. cristatus* and *S. mitis/oralis/infantis* (**C**). Mixed corncob communities in hedgehogs can also contain *Porphyromonas* together with both *S. cristatus* and *S. mitis/oralis/infantis* (D, E), and may also contain cells of family *Pasteurellaceae* as an additional, outer layer on the corncobs (**F**)

A characteristic feature of dental plaque seen in our previous work was the ‘hedgehog’ structure [12]. Operationally, we define a hedgehog as a cluster of *Corynebacterium* filaments with corncobs at their tips. Applying the FISH probe sets to samples from 14 healthy subjects revealed that individual hedgehogs have heterogeneous sets of corncobs (Fig. 2). In some of the hedgehogs, most of the corncob “kernels” (the cocci surrounding the tips of the *Corynebacterium* filaments) were of the same species (Fig. 2A, B). In some cases the species was *S. cristatus* (Fig. 2A) and in other cases it was *S. mitis/oralis/infantis* (Fig. 2B). Other hedgehogs contained corncobs with a mixture of *S. mitis/oralis/infantis* and *S. cristatus* (Fig. 2C). Some hedgehogs had corncobs containing the additional taxa *Porphyromonas* (Fig. 2D–F) and Pasteurellaceae (Fig. 2F). Qualitatively, our results show that dental plaque hedgehogs can be composed of corncobs of varying composition, from either or both of two subgroups of *Streptococcus* and with the presence or absence of members of two additional taxa, *Porphyromonas* and *Pasteurellaceae*. Thus the plaque hedgehog is not a structure of consistent composition but a category of organization in which the filament is constant but the taxonomic composition of the kernels is variable.

### Composition of individual corncobs

**Fig. 3:**
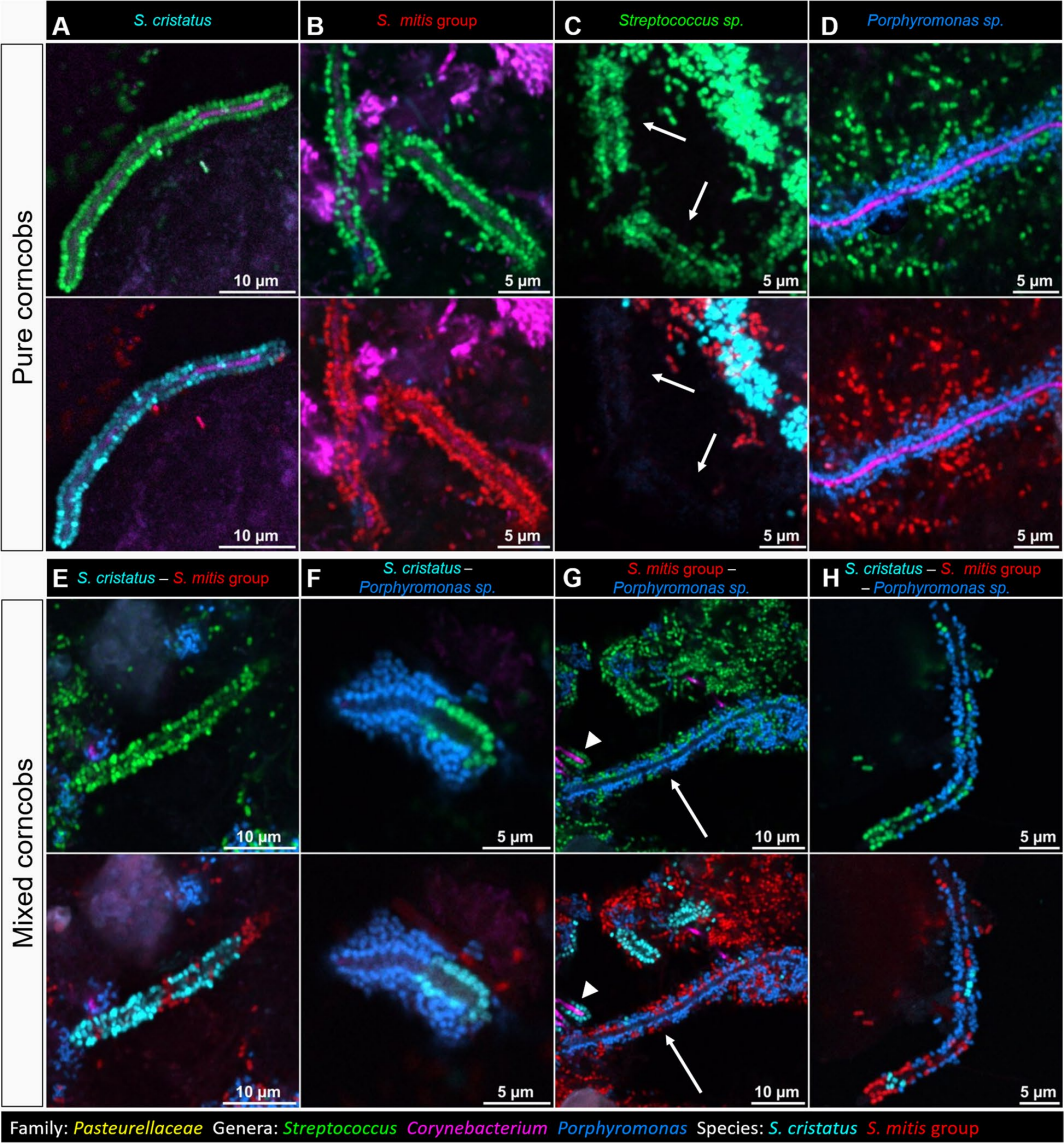
Individual corncobs can contain single or multiple species of *Streptococcus*. Genus probes (top in each pair of images) show the overall structure of corncobs and distinguish between the central filament (*Corynebacterium*) and the surrounding *Streptococcus* (green) or *Porphyromonas* (blue). Staining of the central filament is sometimes absent and in these cases its identity is not confirmed. Species probes (bottom in each pair) distinguish between *S. cristatus* (cyan) and *S. mitis/oralis/infantis* (red). “Pure” corncobs were those in which all the imaged cells around the central filament hybridized to the same probe, targeting *S. cristatus* (**A**), *S. mitis/oralis/infantis* (**B**), a third species of *Streptococcus* not identified with the set of probes used (**C**), or *Porphyromonas* (**D**). “Mixed” corncobs contained more than one type of cells surrounding the central filament: *S. cristatus* and *S. mitis/oralis/infantis* (**E**), *S. cristatus* and *Porphyromonas* sp. (**F**), *S. mitis/oralis/infantis* and *Porphyromonas* sp. (**G**), *S. cristatus, S. mitis/oralis/infantis* and *Porphyromonas* sp. (**H**). Different types are sometimes near each other in the same field of view, e.g., in **G** a mixed corncob of *S. mitis/oralis/infantis* and *Porphyromonas* sp. (arrow), and a pure corncob of *S. cristatus* (△) are observed

Visualizing individual corncobs at higher magnification, we observed that the kernels of a corncob can be composed of a single species or contain mixtures of different species (Fig. 3). The species that were frequently observed were *S. cristatus*, *S. mitis/oralis/infantis*, and *Porphyromonas sp.*, each being observed individually (Fig. 3A–D) and in combinations (Fig. 3 E–H). Interestingly, although we visualized cells of *S. gordonii* in the vicinity of corncobs (Additional file 5), we never observed *S. gordonii* as part of a corncob. We did occasionally observe cells in corncobs that hybridized with the *Streptococcus* genus probe but not with any of the species probes we employed (Fig. 3C). Thus, the individual corncob, like the hedgehog, is not a structure of consistent composition, but is a category of structure in which the taxonomic composition is variable—but the range of variability appears to be limited to a subset of the cocci present in dental plaque.

Corncobs presented themselves in variable conformations within the plaque biofilm. They occurred in groups at the periphery of hedgehog structures, consistent with earlier results showing corncobs forming an outer layer on filament-rich plaque [26]. They also occurred as isolated corncobs embedded in filamentous or non-filamentous plaque. Their length was variable, generally 10–20 μm and as long as 50 μm in a single image. The central filament was sometimes stained with the *Corynebacterium* genus probe (Fig. 3A, D, G) but at other times no staining of the central filament was observed. Variation in intensity of staining of *Corynebacterium* cells is frequently observed (e.g., [12]) and likely results from variation in permeability of the cells to the probe or from variation in ribosome content. Staining is frequently stronger in the center of hedgehog structures than in the corncobs at the periphery (e.g., Fig. 2) and is often variable even within a single corncob (Fig. 3A, B).

### Quantification of corncob types

**Fig. 4:**
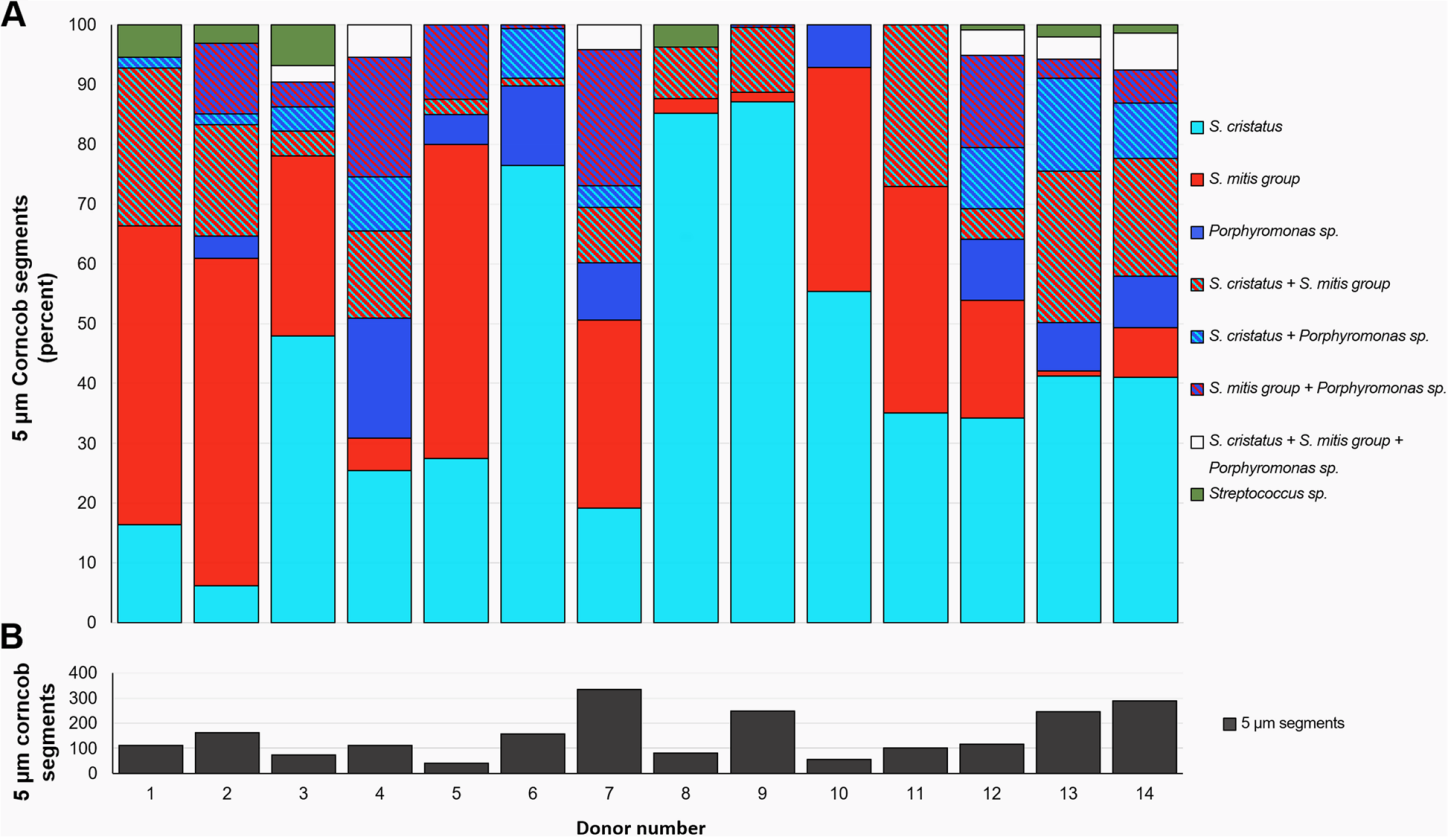
Each donor has a mixed population of corncobs. For each of 14 donors, the imaged corncobs were divided into 5 μm segments and the presence of *S. mitis/oralis/infantis*, *S. cristatus*, other *Streptococcus* spp., and *Porphyromonas* spp. in each segment was recorded. The relative abundance of each type of corncob in each donor is shown (**A**). Black bars indicate the total number of 5 μm corncob segments counted in each donor (**B**). Corncob segments consisting of *S. cristatus* alone constituted 42.3% of the total and a mean of 42.7% (range 6% to 87%) across the 14 individuals. Segments consisting of pure *S. mitis/oralis/infantis* were 19.4% of the total, mean 23.8% (range 0 to 55%) and pure *Porphyromonas* sp. segments were 6.8% of the total, mean 6.1% (range 0–20%). Mixed segments and segments including unidentified *Streptococcus* spp. were 31.6% of the total and mean 27.4% (range 0–50%)

To quantify the relative abundance of different corncob types, we analyzed images of corncobs by dividing the corncobs into segments of 5 μm in length, and classifying the 5 μm segments according to the identity of the layer of cells immediately adjacent to the filament (Fig. 4). For purposes of this quantification, a corncob in longitudinal section was defined operationally as 2 continuous rows of cocci that were at least 5 μm in length on both sides of a core filament—either a visible filament or a gap that was presumed to contain an unstained filament. A corncob in cross section or oblique section was defined as a continuous circle or oval of cocci surrounding a filament or a space presumed to contain an unstained filament. We imaged 10 fields of view (FOV) per probe set and donor, for each of two probe sets and 14 donors, selecting fields of view to image where corncobs were visible through the eyepieces. Not all of these images contained corncobs meeting the criteria for quantification; therefore the total number of FOV in which corncobs were counted averaged 8.3 FOV per probe set and donor (range 3 to 11). The total FOV per donor in which corncobs were counted averaged 17 (range 9–20). From this dataset, we counted a total of 2122 corncob segments with a mean of 152 per donor (range 40–334).

The results of this analysis show that all donors had corncobs of diverse composition (Fig. 4). The imaged corncobs from every donor included both *S. cristatus* and *S. mitis/oralis/infantis*, and images from most donors also included *Porphyromonas*. In about half of the donors, we also detected a small number of corncobs containing cells hybridizing with the *Streptococcus* genus probe but with none of the species probes. About two thirds of the corncob segments contained only a single taxon, either *S. cristatus*, *S. mitis/oralis/infantis*, or *Porphyromonas*, while the remaining one third of segments were mixed (Fig. 4 and Additional file 6). Thus, while the majority of corncob segments were composed of a single taxon, the overall corncob community within each donor was complex.

**Fig. 5:**
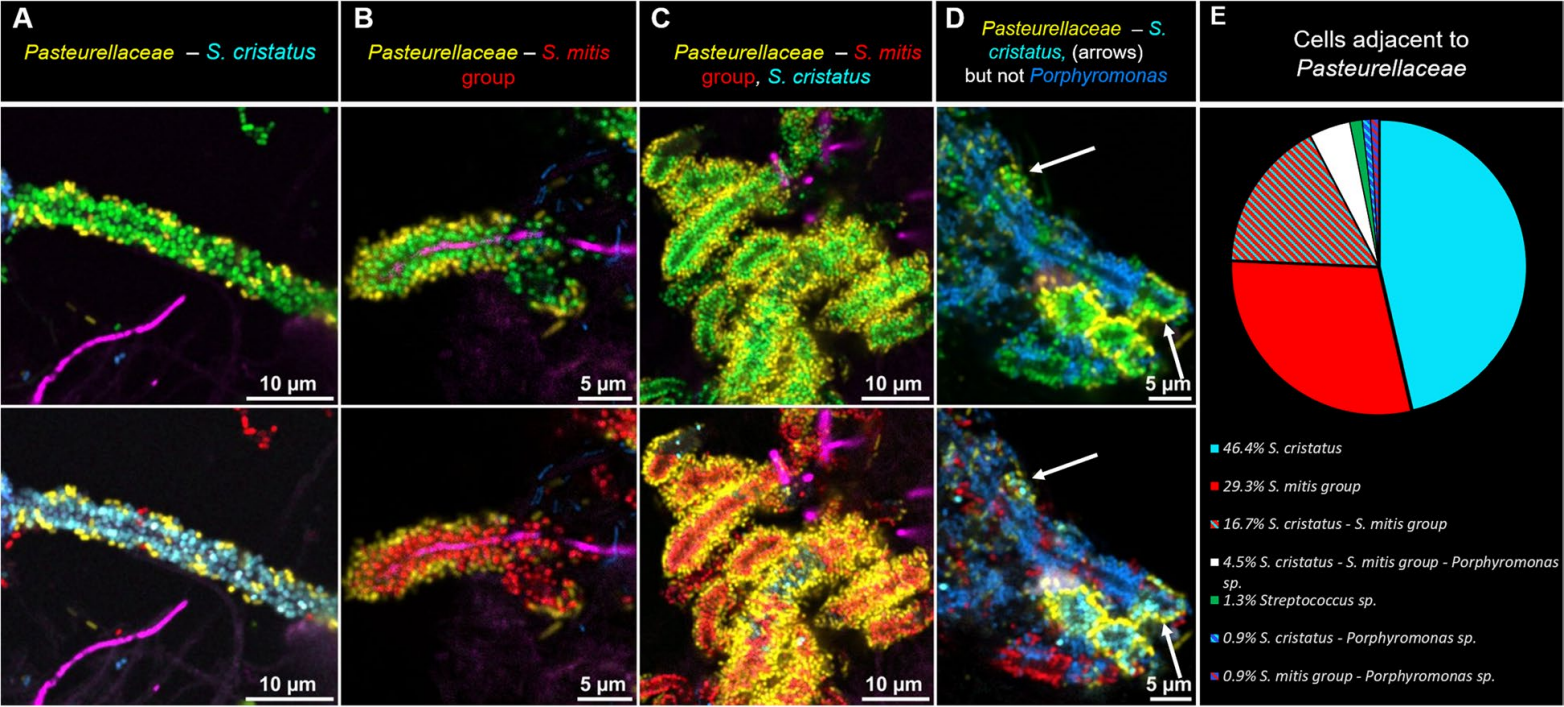
Cells of family *Pasteurellaceae* adhere to *S. mitis/oralis/infantis* and *S. cristatus* but not *Porphyromonas*. In addition to the layer of cells of *Streptococcus* sp. or *Porphyromonas* sp. directly adjacent to the central filament, a second layer of cells from the family *Pasteurellaceae* is sometimes observed on corncobs. We observed these cells attached to corncobs in which the inner layer of cells was *S. cristatus* (**A**), *S. mitis/oralis/infantis* (**B**), or a mixture of *S. cristatus* and *S. mitis/oralis/infantis* (**C**). We also observed a second layer on mixed corncobs that included *Porphyromonas* sp.; in these corncobs the *Pasteurellaceae* cells were adjacent to *Streptococcus* cells but not to the nearby *Porphyromonas* cells (**D**). In the samples analyzed, we did not observe any layer of *Pasteurellaceae* attached to pure *Porphyromonas* corncobs. Among the 5 μm corncob segments counted on the samples hybridized with probe set 1, 21.1% presented a second layer formed by *Pasteurellaceae* (**E**). Of these, close to half (47%) were 5 μm segments in which all the *Streptococcus* cells were *S. cristatus*; 30% of the segments consisted of pure *S. mitis/oralis/infantis*; and the *Streptococcus* population of the remaining 23% was mixed or unidentified

Taxon adjacency relationships in corncobs include not only the relationship between the central *Corynebacterium* filament and the surrounding cocci but also the relationship between this first layer of cocci and the outer layer of cells belonging to the *Pasteurellaceae* [12], of which the representatives abundant in the human mouth are *Haemophilus* and *Aggregatibacter* [1, 7]. To determine whether these cells were found adjacent to all types of inner-layer cocci or only a subset of them, we included a probe for *Pasteurellaceae* in one probe set and detected *Pasteurellaceae* in corncobs from 7 of the 14 donors and in a total of 21.3% of the 1053 corncob segments counted using this probe set (Fig. 5). Results showed that the *Pasteurellaceae* in corncobs associated with both major types of corncob streptococci and also with the unidentified streptococci, in the approximate ratios in which these *Streptococcus* spp. were present in the corncobs overall, but were not found adjacent to corncob *Porphyromonas* (Fig. 5). Thus, our results suggest the possibility of metabolic or binding interactions between *Pasteurellaceae* and both of the two major *Streptococcus* types in corncobs, but not with corncob *Porphyromonas*.

## Discussion

Our findings show that the corncob microhabitat is selective for a subset of the *Streptococcus* species in dental plaque. The most abundant streptococci in dental plaque are members of the *S. mitis/oralis/infantis* cluster and *S. sanguinis*, which together make up about 90% of plaque streptococci. The species *S. cristatus* is a minor component of the genus in plaque as a whole, making up less than 4% of supragingival plaque *Streptococcus* in Human Microbiome Project samples across 148 individuals [7]. Nonetheless, in the 14 individuals studied here, *S. cristatus* was the most abundant *Streptococcus* species in corncobs. Thus, the site-specificity displayed by *S. cristatus* is for a well-defined microhabitat within dental plaque: adhered to *Corynebacterium* filaments as corncobs. The association is not exclusive, however, in that a different *Streptococcus* species, identified by FISH as a member of the *S. mitis/oralis/infantis* group, was almost as abundant as *S. cristatus* in corncobs. Detailed comparison of staining from the *Streptococcus* genus vs. species probes indicated that *S. cristatus* and the *S. mitis/oralis/infantis* group together comprised almost all the *Streptococcus* cells in corncobs; other unidentified *Streptococcus* were rarely present, detected in 1.4% of corncob segments. In particular, *S. gordonii*, a species with overall abundance in supragingival plaque similar to that of *S. cristatus*, was detected in surrounding plaque but not in corncobs. Thus, the corncob represents an interaction between *Corynebacterium* and a limited subset of the pool of plaque *Streptococcus* species. Filament-rich plaque including corncobs makes up only a portion of dental plaque, and in some samples only a modest number of corncobs could be detected. Additional studies with a larger cohort will be needed to make more precise estimates of relative abundance and to determine the identity of the additional streptococci in corncobs.

Members of other genera present in corncobs likewise were adjacent to multiple partners but not all potential partners in plaque. Cells hybridizing with the *Porphyromonas* probe were present in corncobs, either as the only cocci surrounding a filament or sharing a central filament with *S. mitis/oralis/infantis*, *S. cristatus*, or both. The additional outer layer of *Pasteurellaceae* was found adjacent to cells of both *S. mitis/oralis/infantis* and *S. cristatus*, but not *Porphyromonas*. This distribution indicates that the *Pasteurellaceae*-*Streptococcus* relationship in corncobs, like the *Corynebacterium*-*Streptococcus* relationship, is a selective interaction. Although *Pasteurellaceae* spp. associated with two different *Streptococcus* spp. partners, it did not associate with *Porphyromonas* spp. Further study will be needed to determine the mechanistic underpinnings of this spatial selectivity: whether it results from differential binding or differential reproductive success of *Pasteurellaceae* spp. when bound to *Streptococcus* spp. rather than *Porphyromonas* spp., or both.

Although corncob-like structures have been reported to form around other taxa, several lines of evidence suggest that in supragingival plaque the corncob filament is generally *Corynebacterium* spp. In vitro studies [30] have shown that *Fusobacterium nucleatum,* when mixed with *Streptococcus*, can form the central filament of corncob-like structures. However, we have not seen an association of cocci with *Fusobacterium* spp. in natural dental plaque. Our previous results with a probe set targeting different filamentous bacteria in plaque, including *Fusobacterium*, indicated that the corncob association was highly specific to *Corynebacterium* spp. [12]. Although staining of *Corynebacterium* was variable in intensity, the central filament of corncobs, when staining was evident, was always *Corynebacterium*. Other filamentous or elongated taxa such as *Fusobacterium*, *Leptotrichia*, and *Capnocytophaga* were not detected as the central filament even when they were detected in the immediate surroundings of the corncob. A previous study [34] showed associations of streptococci with hyphae of *Candida albicans* in natural plaque. However, *Candida* generally has low abundance in the healthy mouth. In the present study, to focus on species-level identification of streptococci, we omitted probes for the filamentous taxa that our previous study did not detect in corncobs. The quantification results in this study apply to the full population of corncob cocci that we visualized in healthy subjects, whether or not the identity of the central filament could be confirmed.

Our finding of complex but limited taxon composition in corncobs bears on an important question in microbial ecology, namely the question of how a stable, healthy interaction is maintained between a host and its microbiome [35]. Theoretical work predicts that mutualistic interactions tend to fall apart over time, for example because the loss of one of the partners results in the loss of the other, or because one partner ceases to behave as a mutualist and instead becomes a parasite [36]. Such a shift from mutualism to parasitism is more likely if the interaction is highly specific, so that an organism is dependent on a single partner [37]. Bacteria within the densely packed dental plaque biofilm depend on one another for metabolites and signals [19, 38, 39], but the composition of oral microbial communities is characterized by wide fluctuations in the relative abundance of taxa even as the overall community membership remains stable, a pattern known as stationary dynamics [40, 41]. Although the consistent composition and direct cell-cell attachment in corncobs suggests a degree of metabolic dependency of the partners on one another, we observed flexibility in the taxon relationships involved in corncobs, in the sense that several partners were capable of interacting with the central filament and several streptococci could interact with the outer layer of Pasteurellaceae. Thus the spatial relationships we observe in corncobs suggest that each of the microbial participants is capable of interacting with multiple, albeit limited, potential partners, a feature that may encourage the long-term stability of the community.

A related open question in microbial ecology is whether microbial communities assemble with a consistent species composition or, alternatively, with a consistent set of functional genes that can be contributed by a range of different species [42, 43]. It has been proposed, for example, that under conditions common in the mouth (horizontal gene transfer and migration), species identity can be insignificant because genes, rather than species, inhabit niches [44]. Despite the flexibility we observed in the composition of individual corncobs, however, both *S. cristatus* and *S. mitis/oralis/infantis* were observed in corncobs in every donor. At the scale of individual corncobs or corncob segments, these distinct *Streptococcus* species were apparently interchangeable in their ability to bind to the central *Corynebacterium* filament and the exterior shell of *Pasteurellaceae*, yet both types persisted in the plaque community. This persistence suggests that the different taxa possess distinct ecological roles, or that mechanisms exist that stabilize the continued persistence of multiple, functionally redundant taxa within the same microbiome ecosystem. Our data thus indicate that in the corncob microhabitat within the dental plaque biofilm, species composition remains consistent from mouth to mouth.

The heterogeneity of corncob structures has important implications for mechanistic studies such as in vitro co-culture or multi-taxon metabolic modeling of plaque bacteria as a model microbial community. In addition to the *Corynebacterium*-*S. cristatus* relationship, our results show numerous pairs of taxa directly adjacent to one another in corncobs, including all combinations of *S. cristatus*, *S. mitis/oralis/infantis*, *Porphyromonas* spp*.*, and *Corynebacterium* spp. as well as *Pasteurellaceae* with both *S. mitis/oralis/infantis* and *S. cristatus*. Thus, a number of potentially significant taxon-taxon relationships have been identified in this study, and our results suggest that a natural corncob may be modeled not only as a two-taxon relationship but also as a relationship containing three, four, or five partners. The mechanistic underpinnings of the corncob association likely are founded on adhesion of taxa to one another. *C. matruchotii* itself adheres not directly to the tooth surface, but to early colonizers such as *Actinomyces naeslundii* [45]. Among oral streptococci, *S. cristatus* has tufts of fibrils that enable its adhesion to *C. matruchotii* [46] and *S. oralis* subsp. *dentisani* forms fibrils distributed asymmetrically on its surface that likely play a role in adhesion [47]. Whether this subspecies of *S. oralis* is the taxon identified in corncobs by our *S. mitis/oralis/infantis* probe is an important question that could be resolved by the development of in situ sequencing approaches. Although the metabolism of the species visualized here in corncobs has not yet been the subject of extensive in vitro investigation, other oral species within these same genera have been investigated and their taxon-taxon interactions have been shown to change the gene expression and biology of the partners [14, 48, 49]. For example, co-culture of *C. durum* and *S. sanguinis* results in interspecies interactions involving fatty acid metabolism of both partners [45]. Our results enable the selection of taxa for in vitro co-culture studies that are grounded in the frequently adjacent taxa of natural plaque; these are the taxa that are likely to engage in metabolic interactions with physiologically relevant consequences.

Corncobs bear some resemblance to another tight spatial relationship in the oral microbiome, in which ultrasmall *Saccharibacteria* spp. live epibiotically on filamentous Actinobacteria such as *Schaalia odontolytica*, but there are important differences between the two consortia. Oral *Saccharibacteria* are obligate epibionts; they have genome sizes under 0.9 Mb, lack numerous genes essential for a free-living lifestyle, and consequently cannot be cultivated except in co-culture with their host [50–52]. By contrast, corncob taxa (not only *C. matruchotii* but also *S. cristatus*, *S. oralis*, *S. mitis*, and the *Porphyromonas* species most likely to be in corncobs, *P. catoniae* and *P. pasteri*) have genome sizes of approximately 2 Mb and are free-living, capable of growth in pure culture in standard media. In ecological terms, corncob cocci have a fundamental niche (capable of independent growth in standard nutrient-rich conditions) that is broader than their realized niche (generally found adjacent to *Corynebacterium* spp. in corncobs). Thus while the *Saccharibacteria*-host relationship is that of a potentially parasitic epibiont, the corncob represents a consortium of organisms that may prosper in each other’s company but are capable of independent growth.

Several lines of evidence suggest that the taxa participating in corncobs are associated with human health. Recent studies have found *C. matruchotii* and *C. durum* associated with health rather than caries [53–55]. Metabolites produced by *C. durum* have also been found to extend lifespan in the model organism *Caenorhabditis elegans* [56] and *C. durum* elicited no inflammatory response from human gingival and oral mucosal cells, suggesting it is a commensal [57]. *S. cristatus* has been shown to inhibit biofilm formation of the periodontal pathogen *P. gingivalis* by repression of virulence genes [58, 59]. Because of their location towards the outside of plaque, corncob taxa may represent the first organisms that a microbe would encounter when landing on the tooth biofilm; corncobs composed of *S. cristatus* might therefore inhibit colonization of the mouth by this potential pathogen. On the other hand, some mitis group streptococci potentiate the virulence of *C. albicans* [60, 61]. Under what circumstances the species that participate in corncob structures inhibit or enhance the colonization and virulence of pathogens, and the mechanisms by which they do so, is an important question for further research. In studies of the development of plaque on epoxy resin crowns worn by volunteers, the initial plaque was coccus-rich, corncobs were first observed after 3 days, and filament-rich plaque did not occur until approximately 1 week of incubation [25, 26]. These observations might suggest that hedgehog structures and corncobs would be rare in people engaging in daily dental hygiene. In the present study, however, we detected corncobs in all donors and hedgehog structures in most, even though donors were instructed to refrain from oral hygiene for only 24 h and no donor reported going longer than 26 h without tooth brushing. We conclude that hedgehogs and corncobs can be formed in less than 24 h in the dental plaque of healthy individuals, perhaps growing from already-established patches of filament-rich plaque, and that corncob structure and function may play an important role in normal oral microbial ecology and in maintenance of human health.

The unique benefit of the imaging approach we took here is that taxon-taxon spatial relationships can be directly visualized at micrometer scales. The results we report here represent substantial effort in imaging and analysis; to analyze 2122 corncob segments we quantified 232 fields of view, each of which was composed of 5 to 8 unmixed fluorophore channels, for a total of more than 1400 images. Each field of view in our study represented an area of 212 × 212 micrometers and a volume of 4 × 10^−5^ mm^3^. By comparison, each data point in a sequencing study may represent a homogenized sample from a cubic millimeter of biofilm or a swab over several cm of area. Thus, sequencing generates abundant data that can be subjected to sophisticated analysis, but at the cost of losing fine-scale spatial information, and quantitative comparison of our results with the results of sequencing studies is not straightforward because the scale of sampling is so different. Future studies with increasingly complex probe sets and automated image processing may be able to identify hundreds of thousands of cells with micrometer spatial resolution, enabling analysis of the full complexity and micron-scale arrangement of microbes in biofilms.

## Conclusions

Within the complex dental plaque biofilm, corncob structures represent a well-defined microhabitat that is inhabited by a specific subset of the bacteria found in dental plaque as a whole. The spatial adjacency relationships in corncobs indicate that each taxon associates with a limited number of potential partners, but always with more than one potential partner, a feature that may encourage the long-term stability of the community. Our results suggest the general principle that the more precisely a microhabitat can be defined, the more well-defined will be the set of microbial taxa that grow in this habitat. Further work elucidating bacterial nearest-neighbor relationships in oral biofilms may identify additional taxon-taxon associations which could be exploited to enable targeted modulation of these communities for the maintenance of health and the treatment of disease.

## Supplementary Information

**Additional file 1.** Validation of new oligonucleotide probes targeting subsets of the genus *Streptococcus*. For each probe, a full list of oral *Streptococcus* species and their matches and mismatches to the probe is shown (top), along with images showing the intensity of signal imaged after hybridization of pure cultures of cells to the probes. Each newly designed probe was hybridized simultaneously with existing probes targeting genus *Streptococcus* and most Bacteria as controls. 15 pure cultures were hybridized; 8 are shown here and the remaining 7 are shown in Fig. 1. A) Probe Scri995 targeting *S. cristatus*; B) probe Sgor63 targeting *S. gordonii*; C) probe Smit371 targeting *S. mitis* and its close relatives and *S. cristatus*; D) probe Smit651 targeting *S. mitis* and its close relatives. For each probe set, image acquisition and linear unmixing of all cultures were carried out under the same conditions using Zeiss ZEN software. Images were imported into FIJI and the range of display intensities was kept constant for each fluorophore (each column in the figure); for cultures where the fluorescence was dim in all channels, the display range was then additionally adjusted by a constant factor for all images in the row to improve visibility of cells; these rows are marked (*). All probes hybridized with their expected targets and showed negligible cross-hybridization to unexpected targets. (RRX: Rhodamine Red X; TRX: Texas Red X).

**Additional file 2.** List of strains used for FISH on pure cultures.

**Additional file 3.** Ribosomal RNA-targeted oligonucleotide probes used in this study and their combination into probe sets [62–64].

**Additional file 4. **Probe set validation matrices showing hybridization of pure cultures with complete probe sets. For each probe set, image acquisition and linear unmixing of all cultures were carried out under consistent conditions using Zeiss ZEN software. Images were imported into FIJI and the range of display intensities was kept constant for each fluorophore (each column in the figure). In addition to the species probes shown in Fig. 1 and Additional File 1, probes and their targets and fluorophores are as follows: Str405 [62] targeting genus *Streptococcus*, labeled with Rhodamine Red X (RRX); Por1160 [64] targeting the gingivalis group of genus *Porphyromonas*, labeled with Alexa 555; Pas111 [64] targeting family *Pasteurellaceae*, labeled with Dy615; Cor633 [12] targeting genus *Corynebacterium*, labeled with Dy490 (part A) or Atto 620 (part B); probe Cmat175 [12] targeting species *C. matruchotii*, labeled with Atto 655. All probes hybridized with their expected targets and showed negligible cross-hybridization to unexpected targets. (At532: Atto 532; TRX: Texas Red X).

**Additional file 5. **Cells of *Streptococcus gordonii* visualized in supragingival dental plaque. Top panel shows *Streptococcus* spp. at the genus level; bottom panel shows *Streptococcus* at the species level. (A): Cells of *S. gordonii* (arrows) are visualized in the vicinity of corncobs but not attached to them. (B): Cells of *S. gordonii* are also observed in hedgehog structures but not as part of corncobs. (C): a large clump of *S. mitis* group cells with scattered *S. gordonii* cells.

**Additional file 6.** Taxon composition of corncob segments by donor. This is the numerical data underlying the bar chart in Fig. 4.

## Data Availability

The datasets supporting the conclusions of this article are available in Zenodo under doi:10.5281/zenodo.6426109, doi:10.5281/zenodo.6529696, and doi:10.5281/zenodo.6529719.

## Abbreviations

FISH: Fluorescence in situ hybridization
FOV: Field of view
NA: Numerical aperture
