# Supplementary figures and images for: Corncob structures in dental plaque reveal microhabitat taxon specificity
Source: Microbiome. 2022 Sep 5;10:145. doi: 10.1186/s40168-022-01323-x (PMC9446765; doi:10.1186/s40168-022-01323-x)

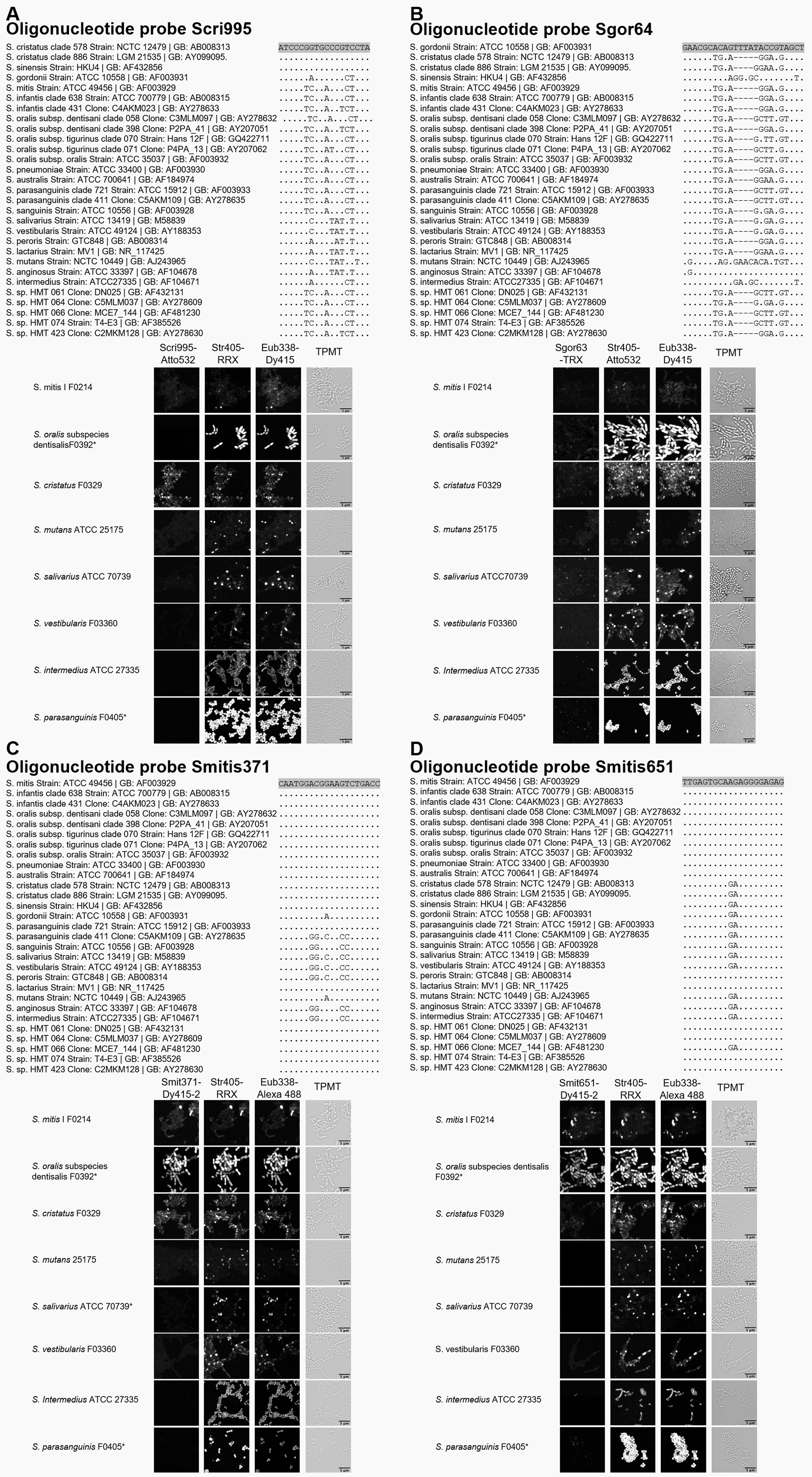

Supplement: Supplementary file 2 — Additional file 1. Validation of new oligonucleotide probes targeting subsets of the genus Streptococcus. For each probe, a full list of oral Streptococcus species and their matches and mismatches to the probe is shown (top), along with images showing the intensity of signal imaged after hybridization of pure cultures of cells to the probes. Each newly designed probe was hybridized simultaneously with existing probes targeting genus Streptococcus and most Bacteria as controls. 15 pure cultures were hybridized; 8 are shown here and the remaining 7 are shown in Fig. 1. A) Probe Scri995 targeting S. cristatus; B) probe Sgor63 targeting S. gordonii; C) probe Smit371 targeting S. mitis and its close relatives and S. cristatus; D) probe Smit651 targeting S. mitis and its close relatives. For each probe set, image acquisition and linear unmixing of all cultures were carried out under the same conditions using Zeiss ZEN software. Images were imported into FIJI and the range of display intensities was kept constant for each fluorophore (each column in the figure); for cultures where the fluorescence was dim in all channels, the display range was then additionally adjusted by a constant factor for all images in the row to improve visibility of cells; these rows are marked (*). All probes hybridized with their expected targets and showed negligible cross-hybridization to unexpected targets. (RRX: Rhodamine Red X; TRX: Texas Red X). [file 40168_2022_1323_MOESM1_ESM.tif]

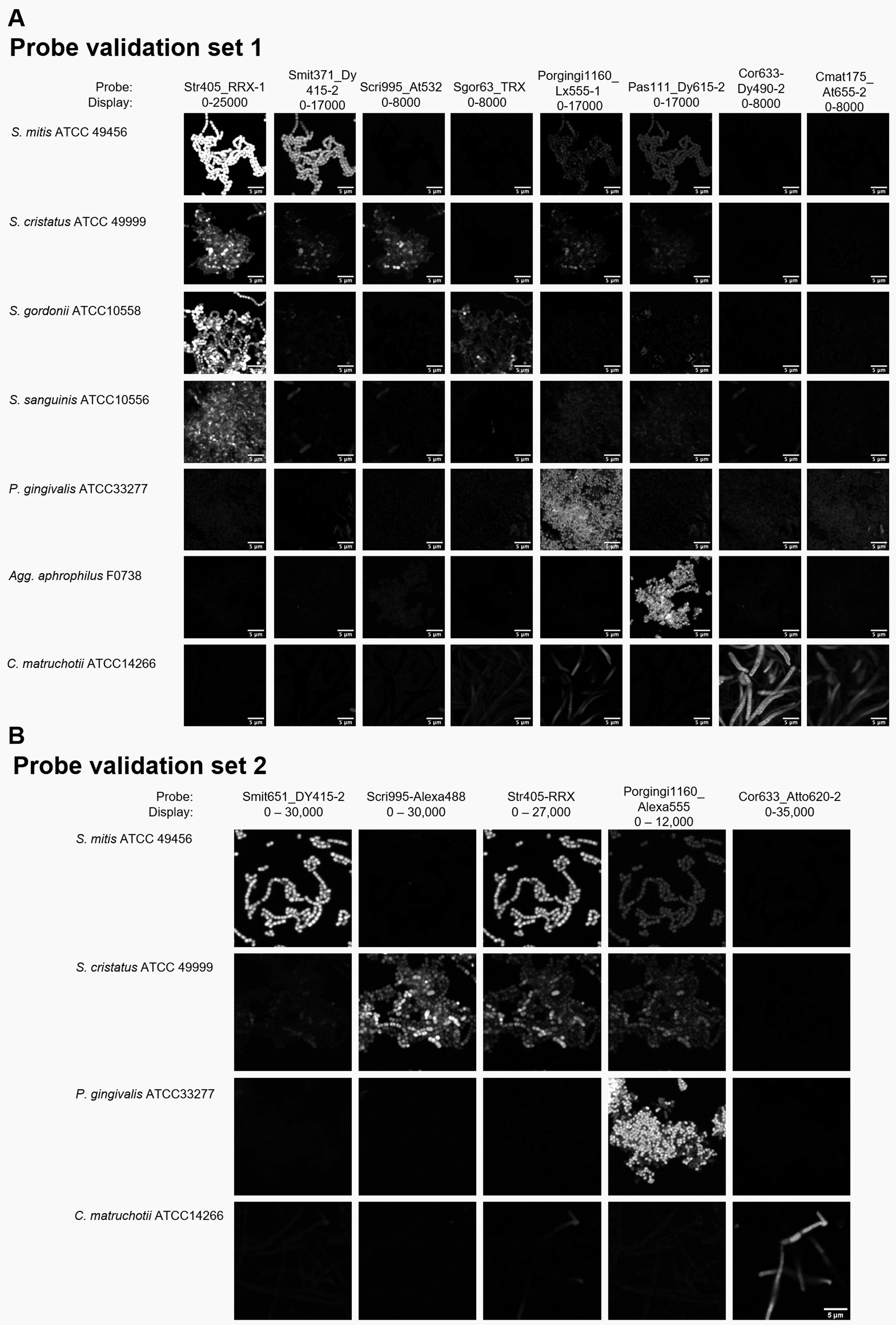

Supplement: Supplementary file 5 — Additional file 4. Probe set validation matrices showing hybridization of pure cultures with complete probe sets. For each probe set, image acquisition and linear unmixing of all cultures were carried out under consistent conditions using Zeiss ZEN software. Images were imported into FIJI and the range of display intensities was kept constant for each fluorophore (each column in the figure). In addition to the species probes shown in Fig. 1 and Additional File 1, probes and their targets and fluorophores are as follows: Str405 [62] targeting genus Streptococcus, labeled with Rhodamine Red X (RRX); Por1160 [64] targeting the gingivalis group of genus Porphyromonas, labeled with Alexa 555; Pas111 [64] targeting family Pasteurellaceae, labeled with Dy615; Cor633 [12] targeting genus Corynebacterium, labeled with Dy490 (part A) or Atto 620 (part B); probe Cmat175 [12] targeting species C. matruchotii, labeled with Atto 655. All probes hybridized with their expected targets and showed negligible cross-hybridization to unexpected targets. (At532: Atto 532; TRX: Texas Red X). [file 40168_2022_1323_MOESM4_ESM.tif]

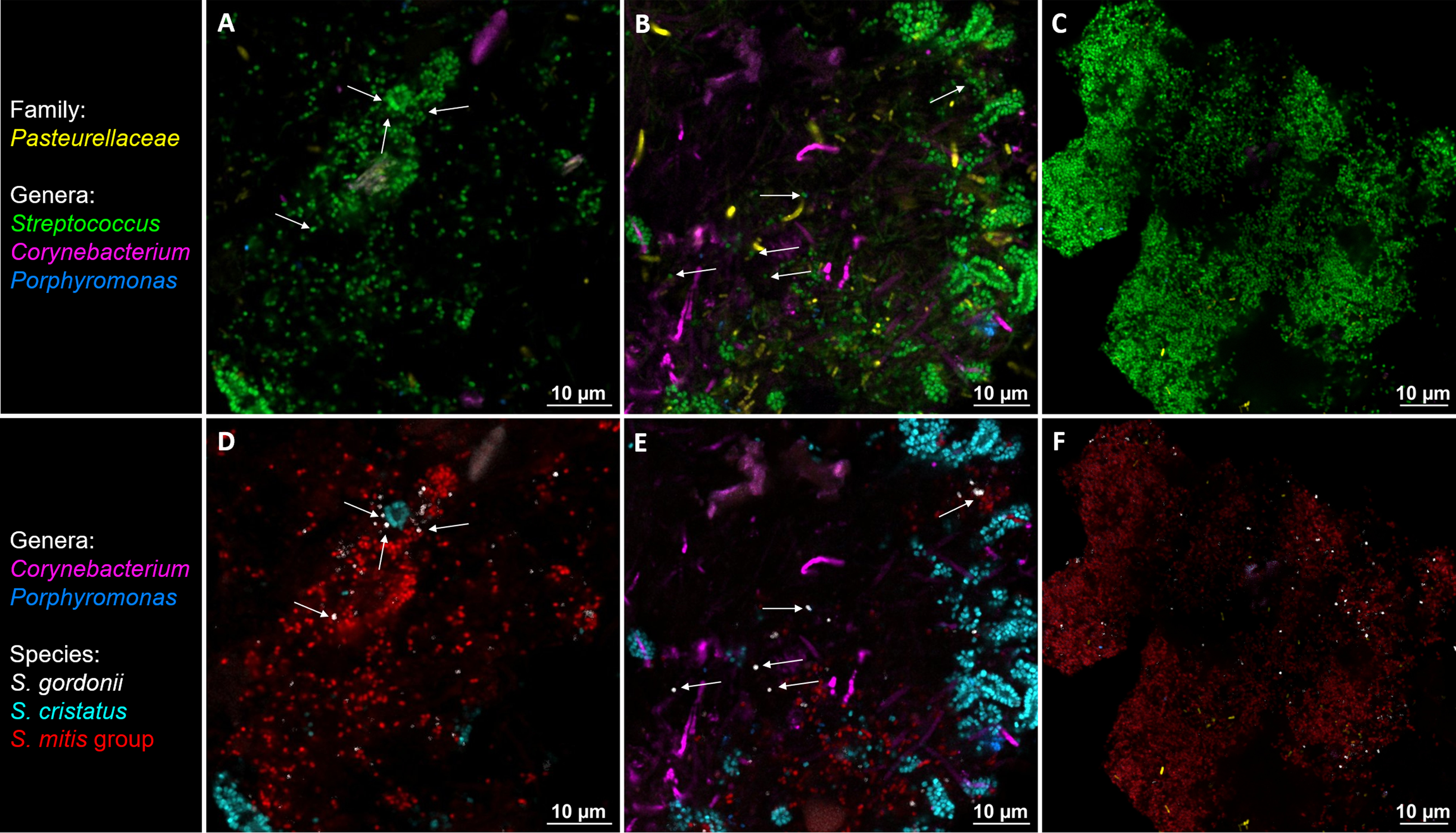

Supplement: Supplementary file 6 — Additional file 5. Cells of Streptococcus gordonii visualized in supragingival dental plaque. Top panel shows Streptococcus spp. at the genus level; bottom panel shows Streptococcus at the species level. (A): Cells of S. gordonii (arrows) are visualized in the vicinity of corncobs but not attached to them. (B): Cells of S. gordonii are also observed in hedgehog structures but not as part of corncobs. (C): a large clump of S. mitis group cells with scattered S. gordonii cells. [file 40168_2022_1323_MOESM5_ESM.tif]
