# Supplementary material for: Corncob structures in dental plaque reveal microhabitat taxon specificity
Source: Microbiome. 2022 Sep 5;10:145. doi: 10.1186/s40168-022-01323-x (PMC9446765; doi:10.1186/s40168-022-01323-x)
Supplement: Supplementary file 3 — Additional file 2. List of strains used for FISH on pure cultures. [file 40168_2022_1323_MOESM2_ESM.docx]

| **Microorganism** | **Strain** | **Previous name** |
| --- | --- | --- |
| *Streptococcus mitis* | ATCC 49456 |  |
| *S. mitis I* | F0214 |  |
| *S. oralis subspecies dentisani* Clade 398 | F0392 | S. mitis II F0392 |
| *S. intermedius* | ATCC 27335 |  |
| *S. oralis 35037* subspecies oralis | ATCC 35037 |  |
| *S. cristatus* | ATCC 49999 |  |
| *S. cristatus* | ATCC 51100 |  |
| *S. cristatus* | F0329 |  |
| *S. salivarius* | ATCC 70739 |  |
| *S. vestibularis* | F03360 |  |
| *S. gordonii* | ATCC 10558 |  |
| *S. mutans* | ATCC 25175 |  |
| *S. pneumoniae* | ATCC 49619 |  |
| *S. sanguinis* | ATCC 10556 |  |
| *S. parasanguinis* clade 441 | F0405 |  |
| *Porphyromonas gingivalis* | ATCC 33277 |  |
| *Corynebacterium matruchotii* | ATCC 14266 |  |
| *Corynebacterium durum* | F0235 |  |
| *Aggregatibacter aphrophilus* | F0738 |  |

Additional File 2. List of strains used for FISH on pure cultures.
