# Supplementary material for: Corncob structures in dental plaque reveal microhabitat taxon specificity
Source: Microbiome. 2022 Sep 5;10:145. doi: 10.1186/s40168-022-01323-x (PMC9446765; doi:10.1186/s40168-022-01323-x)
Supplement: Supplementary file 4 — Additional file 3. Ribosomal RNA-targeted oligonucleotide probes used in this study and their combination into probe sets [62–64]. [file 40168_2022_1323_MOESM3_ESM.docx]

| **Probe** | **Fluorophore** | **Target organism** | **Probe sequence (5' - 3')** | **Reference** |
| --- | --- | --- | --- | --- |
| **Test for specificity of *Streptococcus* species-level probes** | | | | |
| ***S. cristatus* probe** | | |  |  |
| Scri995 | Atto 532-2 | *S. cristatus, S. sinensis* | 5' TAGGACGGGCACCGGGAT 3' | This paper |
| Str405 | Rhodamine Red X | Genus *Streptococcus* | 5' TAGCCGTCCCTTTCTGGT 3' | Paster et al.1998 [62] |
| Eub338 | Dy415 | Domain Bacteria | 5' GCTGCCTCCCGTAGGAGT 3' | Amann et al. 1990 [63] |
| ***S. gordonii* probe** | | | | |
| Sgor63 | Texas Red X - 2 | *S. gordonii, S. anginosus* | 5' AGCTACGGTATAAACTGTGCGTTC 3' | This paper |
| Str405 | Atto 532-2 | Genus *Streptococcus* | 5' TAGCCGTCCCTTTCTGGT 3' | Paster et al. 1998 [62] |
| Eub338 | Dy415 | Domain Bacteria | 5' GCTGCCTCCCGTAGGAGT 3' | Amann et al. 1990 [63] |
| ***S. mitis* probe 1** | |  |  |  |
| Smit371 | Dy415-2 | Streptococcus mitis group (*S. mitis, S. oralis, S. infantis, S. australis, S. pneumoniae*); *S. cristatus* | 5’ GGTCAGACTTCCGTCCATTG 3’ | This paper |
| Str405 | Rhodamine Red X | Genus *Streptococcus* | 5' TAGCCGTCCCTTTCTGGT 3' | Paster et al. 1998 [62] |
| Eub338 | Alexa 488 | Domain Bacteria | 5' GCTGCCTCCCGTAGGAGT 3' | Amann et al. 1990 [63] |
| ***S. mitis* probe 2** | |  |  |  |
| Smit651 | Dy415-2 | Streptococcus mitis group (*S. mitis, S. oralis, S. infantis, S. australis, S. pneumoniae*) | 5’ CCCCTCTTGCACTCAA 3’, | This paper |
| Str405 | Rhodamine Red X | Genus *Streptococcus* | 5' TAGCCGTCCCTTTCTGGT 3' | Paster et al. 1998 [62] |
| Eub338 | Alexa 488 | Domain Bacteria | 5' GCTGCCTCCCGTAGGAGT 3' | Amann et al. 1990 [63] |
| **Probe Set 1** |  |  |  |  |
| Smit371-Dy415-2 | Dy415 | Streptococcus mitis group (*S. mitis, S. oralis, S. infantis, S. australis, S. pneumoniae*); *S. cristatus* | 5’ GGTCAGACTTCCGTCCATTG 3’ | This paper |
| Cor633-Dy490-2 | Dy490 | Genus *Corynebacterium* | 5' AGTTATGCCCGTATCGCCTG 3' | Mark Welch et al. 2016 [12] |
| Scri995-At532-2 | Atto532 | *S. cristatus, S. sinensis* | 5' TAGGACGGGCACCGGGAT 3' | This paper |
| Por1160-Lx555-1 | Alexa555 | Genus *Porphyromonas* | 5' CCTCACGCCTTACGACGG 3' | Valm et al. 2011 [64] |
| Str405-RRX | Rhodamine Red X | Genus *Streptococcus* | 5' TAGCCGTCCCTTTCTGGT 3' | Paster et al. 1998 [62] |
| Sgor63-TRX-2 | Texas Red X | *S. gordonii* | 5' AGCTACGGTATAAACTGTGCGTTC 3' | This paper |
| Pas111-Dy615-2 | Dy615 | Family *Pasterurellaceae* | 5' TCCCAAGCATTACTCACC 3' | Valm AM et al. 2011 [64] |
| Cmat175-At655-2 | Atto655 | *C. matruchotii* | 5' ACTAAACCATGGTCCTATCCG 3' | Mark Welch et al. 2016 [12] |
| **Probe Set 2** | | | | |
| Smit651-Dy415-2 | Dy415 | Streptococcus mitis group (*S. mitis, S. oralis, S. infantis, S. australis, S. pneumoniae*) | 5’ CCCCTCTTGCACTCAA 3’, | This paper |
| Scri995-Alexa 488 | Alex488 | *S. cristatus, S. sinensis* | 5' TAGGACGGGCACCGGGAT 3' | This paper |
| Por1160-Lx555-1 | Alexa555 | Genus *Porphyromonas* | 5' CCTCACGCCTTACGACGG 3' | Valm et al. 2011 [64] |
| Str405-RRX | Rhodamine Red X | Genus *Streptococcus* | 5' TAGCCGTCCCTTTCTGGT 3' | Paster et al. 1998 [62] |
| Cor633-Atto620-2 | Atto620 | Genus *Corynebacterium* | 5' AGTTATGCCCGTATCGCCTG 3' | Mark Welch et al. 2016 [12] |
| **Probe Set 3** | | | | |
| Smit651-Dy415-2 | Dy415 | Streptococcus mitis group (*S. mitis, S. oralis, S. infantis, S. australis, S. pneumoniae*) | 5’ CCCCTCTTGCACTCAA 3’, | This paper |
| Scri995-Atto 532-2 | Atto532 | *S. cristatus, S. sinensis* | 5' TAGGACGGGCACCGGGAT 3' | This paper |
| Por1160-Lx555-1 | Alexa555 | Genus *Porphyromonas* | 5' CCTCACGCCTTACGACGG 3' | Valm et al. 2011 [64] |
| Str405-RRX | Rhodamine Red X | Genus *Streptococcus* | 5' TAGCCGTCCCTTTCTGGT 3' | Paster et al. 1998 [62] |
| Cor633-Atto620-2 | Atto620 | Genus *Corynebacterium* | 5' AGTTATGCCCGTATCGCCTG 3' | Mark Welch et al. 2016 [12] |

Additional file 3. Ribosomal RNA-targeted oligonucleotide probes used in this study and their combination into probe sets.
